# Successful treatment of recalcitrant cutaneous lichen planus and unusual variants with upadacitinib: A case series and a literature review of systemic Janus kinase inhibitors use in cutaneous lichen planus and lichen planopilaris

**DOI:** 10.1016/j.jdcr.2025.01.014

**Published:** 2025-02-06

**Authors:** Megan E. McNamara, Leonardo Tjahjono

**Affiliations:** a Georgetown Lombardi Comprehensive Cancer Center, Georgetown University School of Medicine, Department of Oncology, Washington, District of Columbia; b Department of Dermatology, The George Washington University School of Medicine and Health Sciences, Washington, District of Columbia

**Keywords:** hypertrophic lichen planus, Janus kinase 1 inhibitor, lichen planopilaris, lichen planus pigmentosus, lichen planus, lichenoid dermatitis, literature review, upadacitinib

## Introduction

Cutaneous lichen planus (LP) is a chronic inflammatory condition that classically presents as polygonal, flat-topped, pruritic, and violaceous papules. However, there are other rare and unusual variants, including LP pigmentosus, hypertrophic LP, and lichen planopilaris (LPP).^1^ Treatment typically requires combination of topical and systemic regimens, including topical corticosteroids, topical calcineurin inhibitors (TCI), systemic antibiotics, and classical immunosuppressants.^1^ However, there are not currently any FDA-approved treatments and patients can remain recalcitrant despite the use of combination treatments. Janus kinase (JAK) inhibitors are emerging as an effective class of treatments. We report a case series of successful treatment of recalcitrant cutaneous LP and its variants with upadacitinib (UPA) and literature review of UPA use for cutaneous LP and its variants.

## Case series

All of the active and pruritic eruptions in this cohort were biopsied and they all showed lichenoid dermatitis with varying degrees of basal vacuolar degeneration, which are supportive of cutaneous LP and its variants. None of the patients were on systemic medications that are classically thought of as culprits of lichenoid drug eruptions and none have comorbidities, such as hepatitis C. All of the patients showed positive response and UPA was well tolerated. Summary of our cohorts and literature review are summarized in Table I.

**Table I:** Summary of this case series and cases of successful use of systemic Janus kinase inhibitors on cutaneous LP and LPP

Gender, age	Type of LP	Previous treatments	JAK I regimen (mg dose, months)	Outcome
M, 50	LP pigmentosa	TCS, TCI, HCQ	UPA 30 for 2 weeks, 15 for 3.5 moths, 15 twice a week for 5 months	Cleared after 4 months only with PIH, sustained clearance after lowered dose on 9 month follow-up
F, 20	Hypertrophic LP	TCS, ICS, tazarotene cream	UPA 15, ongoing	Significant improvement after 4 months
F, 50	LPP	TCS, TCI, HCQ, MMF, MTX, OABX	UPA 30 mg for 8 weeks, then 15 mg, ongoing	Significant improvement in pruritus and sustained even with lower dose. Noted mild hairgrowth.
F, 40	Generalized cutaneous LP	TCS, TCI, HCQ, MMF	UPA 30 mg for 2 weeks, 15 mg for 4.5 months, 15 mg 3 times weekly for 7 months	Significant improvement with resolution of lesions, sustained improvement even with lower dose
F, 26	Generalized cutaneous LP	ICS, Photo	UPA 15,4	Marked improvement, generally clear with treatment
M, 43	Generalized cutaneous LP	Photo, ICS, Acitretin	UPA 15,ongoing	Marked improvement, generally clear with treatment
F, 25	Generalized cutaneous LP	TCS	UPA 15,ongoing	Marked improvement, generally clear with treatment
F, 56	Generalized cutaneous LP	TCS, Photo, Intramuscular CS	UPA 15,ongoing	Marked improvement, generally clear with treatment
F, 35	Generalized cutaneous LP	TCS, TCI, Intramuscular CS	UPA 15, ongoing	Marked improvement, generally clear with treatment
F, 46	Generalized cutaneous LP	OABX, TCS, OCS, MTX	UPA 15,1.5	Resolution of pruritus
M, 14	LPP	IFX, TCS, ICS, OCS, MTX, HCQ, OMX	UPA 30,7	Regrowth of hair, acne, transient liver enzyme increase; Crohn's disease controlled
F, 51	LPP	ICS, ISO, TMX, Finasteride	UPA 30,3	Regrowth of hair
M, 50	LPP	TCS, ICS, HCQ, ISO, TMX, Pioglitazone, OABX	UPA 30,9	
F, 30	LPP	TCS, ICS, OCS, HCQ, ISO, OABX, CSA	UPA 30,4	Regrowth of hair
F, 42	LPP	TCS, ICS, MTX, HCQ, OMX, ISO, Mesotherapy with platelet-rich plasma	UPA 15,7	Regrowth of hair. Acne
F, 48	Blaschkoid LP	TCS, OCS, MTX, Photo	UPA 15, ongoing	Resolution of pruritus at month 1, Complete resolution of the disease at month 6, reflare when dose lowered to every other day
F, 45	Hypertrophic LP	TCS, Roflumilast	Deu 6, ongoing	Improvement after 1.5 months of therapy and resolution afte 4.5 months. Concommitant use of clobetasol
F, 52	Generalized cutaneous LP	TCS	Deu 6, ongoing	Improvement with reduction of BSA to 20%. Treated concomittantly with TCS and ruxolitinib cream
M, 38	LP pemphigoides	OCS,MMF,MTX,AZA	Tofa 10	Therapeutic response after 2 months of treatment
F, 52	Ulcerative plantar LP	ICS, MTX, CSA, inteamuscular CS	Tofa 10, 3	Resolution and no recurrence after stopping with only tacrolimus ointment maintenance
F, 28	Generalized cutaneous LP	TCS, OCS, hcq	Tofa 20, ongoing	Resolution after 1 month, and sustained on 3-month follow up
M, 50	Hypertrophic LP	TCS, ICS, HCQ, Adalimumab	Tofa 20, ongoing	Improvement after 6 weeks and resolution after 3 month
M, 51	Hypertrophic LP	TCS, OCS, ICS, MTX, MMF, Retinoids, CSA, AZA, Photo, Ixekizumab, Guselkumab	Tofa 10	Drastic improvement
M	LPP	TCS, ICS, TMX, HCQ, PIO, OCS	Tofa 15, 10	Concomitant treatment with ICS. Change in LPPAI -73%
M	LPP	PIO, MMF	Tofa 10, 17	Change in LPPAI -68%
M	LPP	TCS, ICS, ABX, OCS, HCQ, PIO, finasteride	Tofa 10,3	No improvement
F	LPP	TCS, ICS, ABX, HCQ	Tofa 10, 9	Concomitant treatment with ICS. Change in LPPAI -32%
F	LPP	TCS, ICS, ABX, HCQ, finasteride	Tofa 10, 6	Concomitant treatment with ICS and hcq. Change in LPPAI -94%
F	LPP	TCS, ABX, Finasteride	Tofa 10, 7	Concomitant treatment with ICS and TCI. No improvement
F	LPP	ICS	Tofa 15, 19	Flared after stopping tofacitinib
M	LPP	TCS, ABX, HCQ, PIO, finasteride, MMF	Tofa 10,16	Concomitant treatment with hcq. Change in LPPAI -69%
F	LPP	ICS, abx, finasteride	Tofa 10, 10	Change in LPPAI -30%
F	LPP	TCS, ABX	Tofa 10, 2	Change in LPPAI -5%
F, 65	Generalized cutaneous LP	Photo, TCS	Abro 100,2	Significant improvement in weeks, then clear with treatment
F, 25	Generalized cutaneous LP	TCS, ICS	Abro 100, 3.5	Significant improvement in weeks, then clear with treatment
F, 78	Generalized cutaneous LP and hypertrophic LP	TCS, OCS	Bari 2, 4	No data
M, 71	Generalized cutaneous LP and hypertrophic LP	TCS, TCI	Bari 2, 4	Change in total body lesion count −82, change in BSA -8%, change in PP NRS -6, change in pain NRS-6, time to respond: 2 weeks
F, 64	Generalized cutaneous LP and oral LP	TCS	Bari 2, 4	Change in total body lesion count −29, change in BSA -0.15%, change in PP NRS -8, change in pain NRS -8, time to respond: 3 weeks
F, 48	Generalized cutaneous LP and hypertrophic LP	TCS, ICS, OCS	Bari 2, 4	Change in total body lesion count −4, change in BSA -0.1%, change in PP NRS -4.5, change in pain NRS N/A, time to respond: 1 weeks
F, 82	Generalized cutaneous LP and hypertrophic LP	TCS	Bari 2, 4	Change in total body lesion count −110, change in BSA -0.4.5%, change in PP NRS -7, change in pain NRS -7, time to respond: 2 weeks
F, 38	Generalized cutaneous LP	TCS, OCS	Bari 2, 4	Change in total body lesion count −275, change in BSA -5%, change in PP NRS -7, change in pain NRS -7, time to respond: 2 weeks
F, 67	Generalized cutaneous LP	TCS, OCS	Bari 2, 4	Change in total body lesion count −102, change in BSA -2.8%, change in PP NRS -8, change in pain NRS -8, time to respond: 1 weeks
F, 47	Generalized cutaneous LP	TCS	Bari 2, 4	Change in total body lesion count −570, change in BSA -9.8%, change in PP NRS -6, change in pain NRS -8, time to respond: 1 weeks
F, 56	Generalized cutaneous LP	ICS, OCS, intramuscular CS	Bari 2, 4	Change in total body lesion count −150, change in BSA -5%, change in PP NRS -7, change in pain NRS -7, time to respond: 2 weeks
F, 69	Generalized cutaneous LP and oral LP	TCS, MTX	Bari 2, 4	Change in total body lesion count −25, change in BSA -1.5%, change in PP NRS -1, change in pain NRS -3, time to respond: 2 weeks
F, 67	Generalized cutaneous LP	TCS	Bari 2, 4	Change in total body lesion count −163, change in BSA -3.4%, change in PP NRS -6, change in pain NRS -6, time to respond: 2 weeks
F, 76	Generalized cutaneous LP and hypertrophic LP	TCS	Bari 2, 4	Change in total body lesion count −15, change in BSA -2.5%, change in PP NRS -3, change in pain NRS -2, time to respond: 8 weeks

*Abro*, Abrocitinib; *AZA*, azathioprine; *Bari*, baricitinib; *BSA*, body surface area; *CSA*, cyclosporine; *Deu*, deucravacitinib; *HCQ*, hydroxychloroquine; *ICS*, intralesional corticosteroids; *IFX*, infliximab; *ISO*, isotretinoin; *LPPAI*, lichen plano pilaris activity index; *MTX*, methotrexate; *NRS*, numerical rating score; *OABX*, oral antibiotics; *OCS*, oral corticosteroids; *OMX*, oral minoxidil; *PHOTO*, phototherapy; *PIO*, pioglitazone; *TCI*, topical calcineurin inhibitor; *TCS*, topical corticosteroids; *TMX*, topical minoxidil; *Tofa*, tofacitinib.

## Case 1

**Fig 1:**
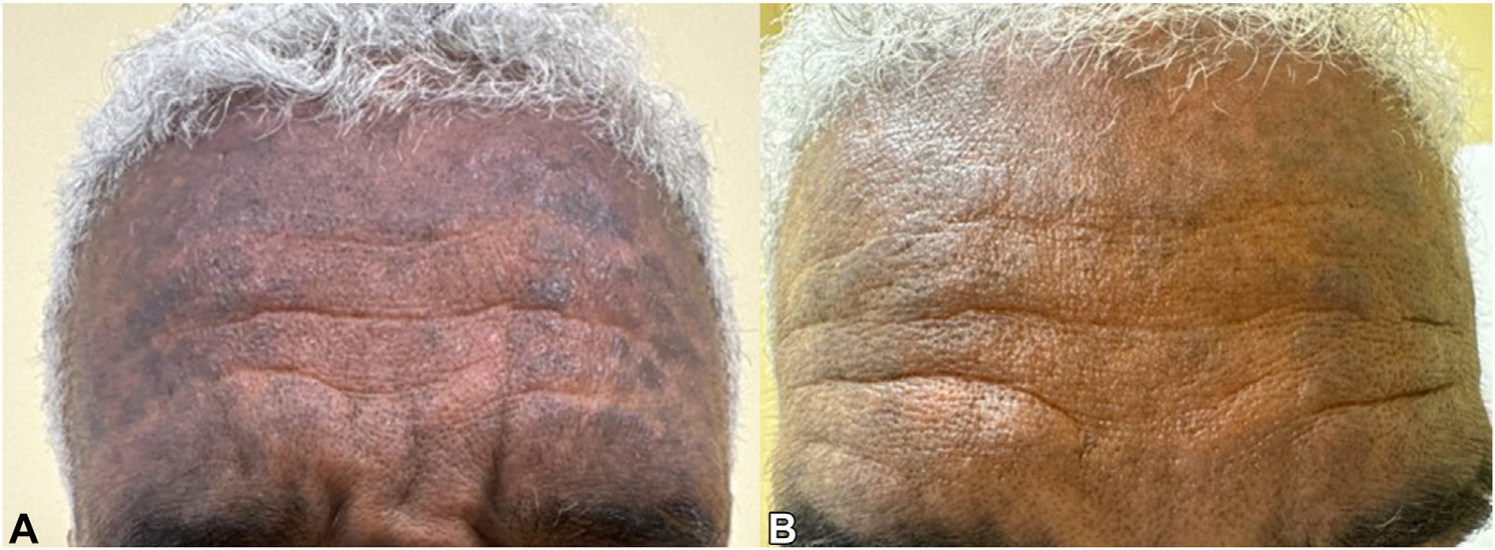
**A** and **B**, Lichen planus (LP) pigmentosa presenting with reticulated, gray-violaceous patches plaques with background ill-defined erythematous patch on the forehead (Fig 1, *A*). The patches and background erythema substantially improved after 4 m use of upadacitinib (UPA) (Fig 1, *B*) and sustained after decrease dosing to twice a week on a 9-m follow up.

**Fig 2:**
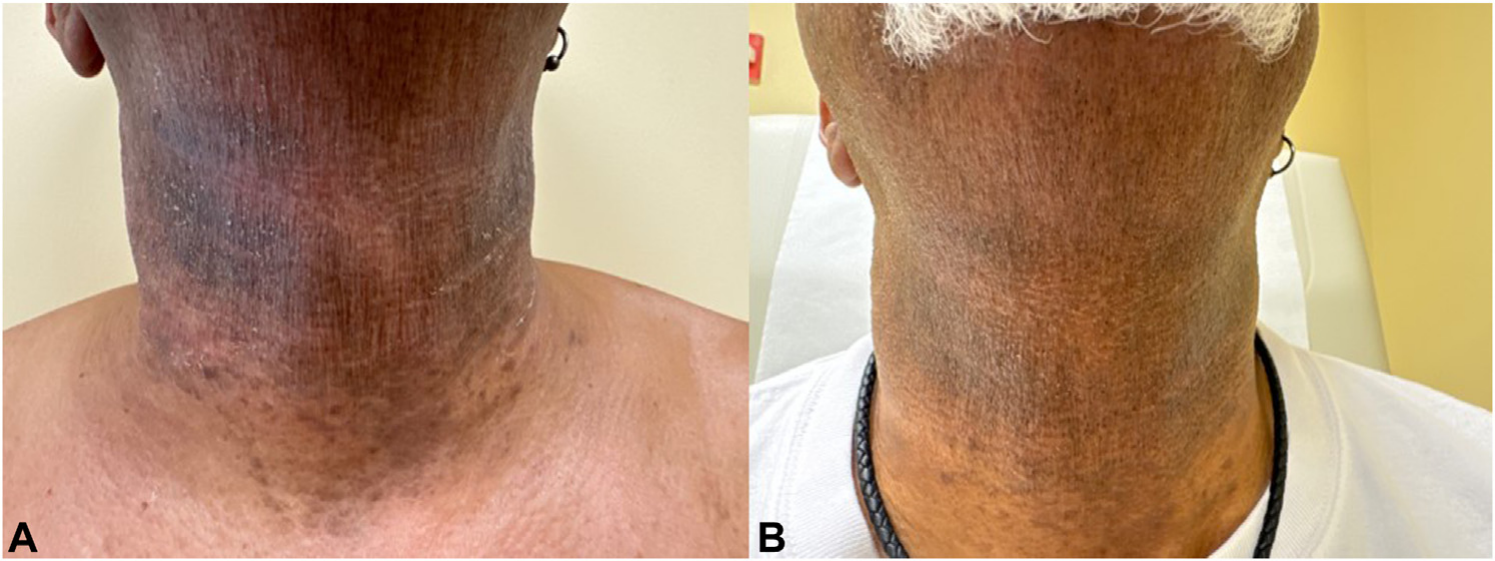
**A** and **B**, Lichen planus (LP) pigmentosa presenting with reticulated, gray-violaceous patches plaques with background ill-defined erythematous patch on the neck (Fig 2, *A*). The patches and background erythema substantially improved after 4 m use of upadacitinib (UPA) (Fig 2, *B*).

A male in his 50s with past medical history (PMH) of well controlled hyperlipidemia and mild hypertension presented with pruritic, reticulated, gray-violaceous patches plaques with background ill-defined erythematous patch on the forehead and neck (Figs 1, *A* and 2, *A*) for 4 m duration. The initial pruritus numerical rating scale (PNRS) was 8 out of 10. Biopsies were obtained from the forehead and the neck, supportive for LP pigmentosus. Topical triamcinolone 0.1% ointment and tacrolimus 0.1% ointment were used in alternating fashion with hydroxychloroquine (hcq) 200 mg two times a day that was trialed for 4 m with no improvement. Due to recalcitrance, we switched hcq to UPA 30 mg daily for 2 weeks and 15 mg daily for 4 m with samples provided by manufacturer. The PNRS improved to 1/10, with improvement in the background erythema and fading of gray-violaceous patches. The current predominant lesion is now post-inflammatory hyperpigmentation (PIH) (Fig 2, *A* and *B*). Use of UPA was approved by insurance and the improvement was sustained on 9-m follow up even with decreased dosing to 15 mg for two times a week. The patient was able to discontinue all topical treatments while maintaining the positive response.

## Case 2

**Fig 3:**
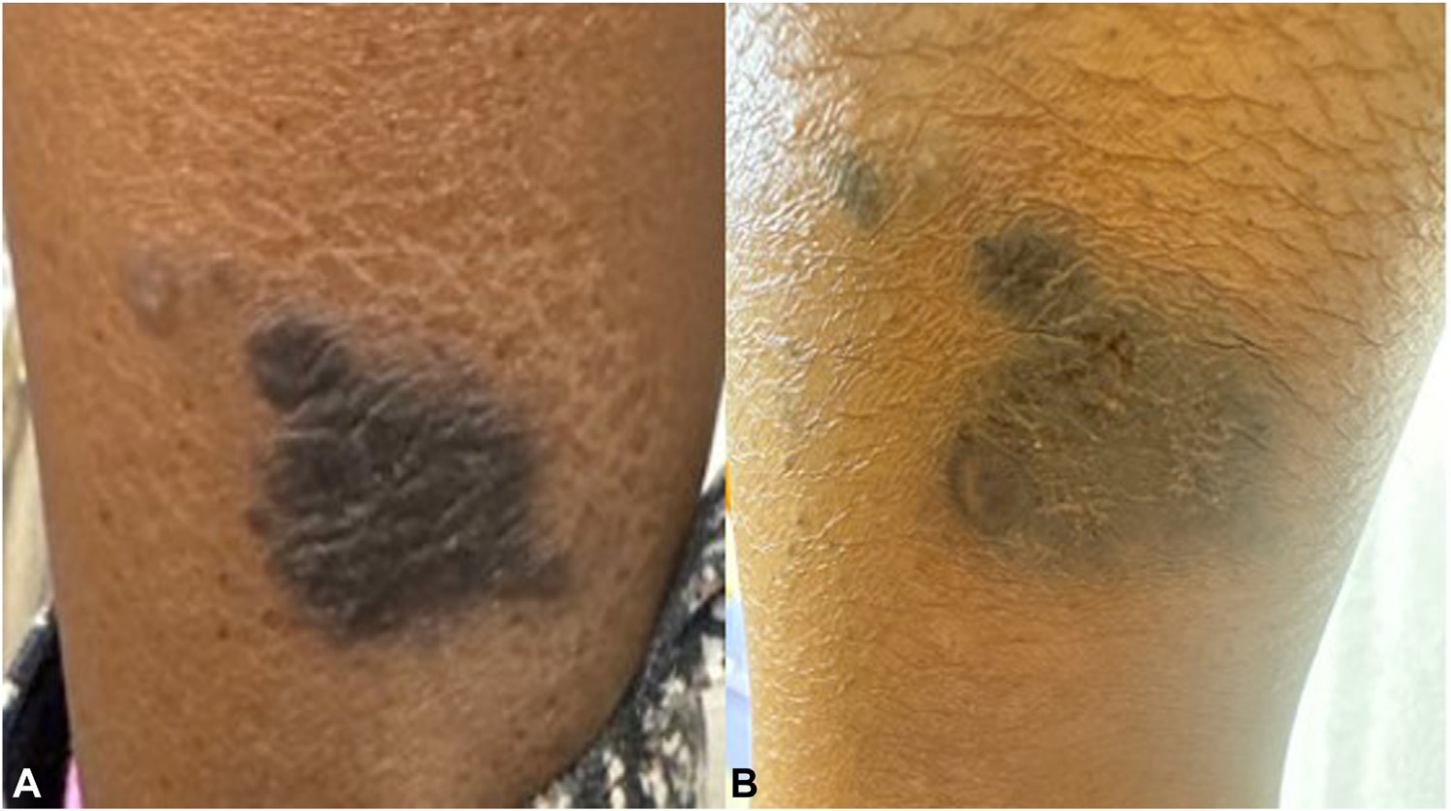
**A** and **B**, Hypertrophic lichen planus (LP) presenting with thick, hyperkeratotic, violaceous plaques (Fig 3, *A*). Four months of upadacitinib (UPA) us improved the thickness and pruritus; the majority of the lesion is dark brown patch, representing post-inflammatory hyperpigmentation (PIH) (Fig 3, *B*).

A female in her 20s with no PMH presented with a pruritic (PNRS 9/10) thick, flat-topped, hyperkeratotic, violaceous plaque on the left lateral ankle (Fig 3, *A*) for 3 years duration. The patient had been trying triamcinolone 0.1% ointment prior to the initial visit. A biopsy was taken from the lesion, supporting the diagnosis of hyperkeratotic LP. Clobetasol 0.05% ointment twice a day and Tazarotene 0.1% cream once a day, along with a series of monthly intralesional triamcinolone of 10 mg/mL were tried for 6 m with no improvement. UPA 15 mg daily was added given the impact of the lesion on her quality of life (QoL); intralesional triamcinolone was discontinued when UPA was started, while the two topical treatments were continued. After 4 m, the plaque and hyperkeratosis significantly improved (Fig 3, *B*) and PNRS improved to a manageable 2/10 and the majority of the lesion is now PIH. The patient is continuing with the current regimen.

## Case 3

**Fig 4:**
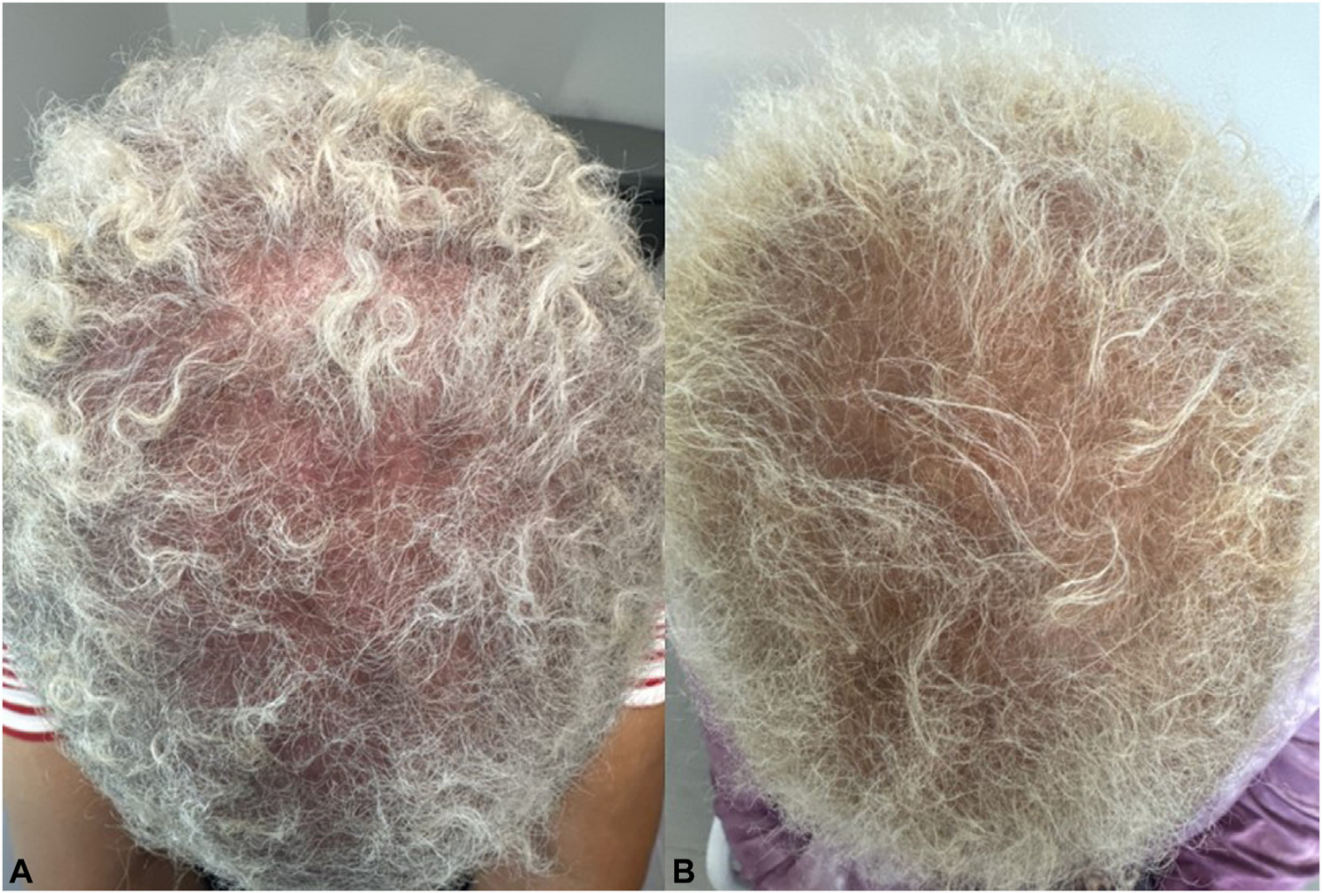
**A** and **B**, Lichen planopilaris (LPP) presenting with innumerable, perifollicular, scaly, keratotic plugs, on diffused background ill-defined erythema (Fig 4, *A*), while still experiencing increased hair loss. The pruritus, hair loss, background erythema, and perifollicular scale substantially improved following 6 m use of upadacitinib (UPA) (Fig 4, *B*)

A female in her 60s with PMH significant for mild hyperlipidemia presented with an innumerable, pruritic (PNRS 8/10), perifollicular, scaly, keratotic plugs, on diffused background ill-defined erythema (Fig 4, *A*). Trichoscopy showed a larger magnification of the perifollicular, keratotic plus, and surrounding erythema. Biopsy was taken from one of the inflamed hair follicles, supporting the diagnosis of LPP. Prior to initial visit, she tried various combination of topical corticosteroid and TCI solution with oral minoxidil 2.5 mg daily and systemic immunosuppressants, such as hcq, mycophenolate mofetil (MMF), methotrexate (MTX), and various systemic antibiotics for months with nonsatisfactory improvement. She continued to experience significant hair loss despite the treatment combinations, so the immunosuppressants and the systemic antibiotics were discontinued. UPA 30 mg daily was added to regimen of TCI solution, oral minoxidil 2.5 mg daily, and hcq 200 mg twice a day as a combination for 8 weeks. Then, UPA dosing was decreased to 15 mg UPA daily for 6 m. She noted a cessation of hair shedding and improvement of PNRS to a manageable 2/10; she also noted mild hair growth. She was able to discontinue TCI and her LPP is stable on 15 mg UPA daily.

## Case 4

**Fig 5:**
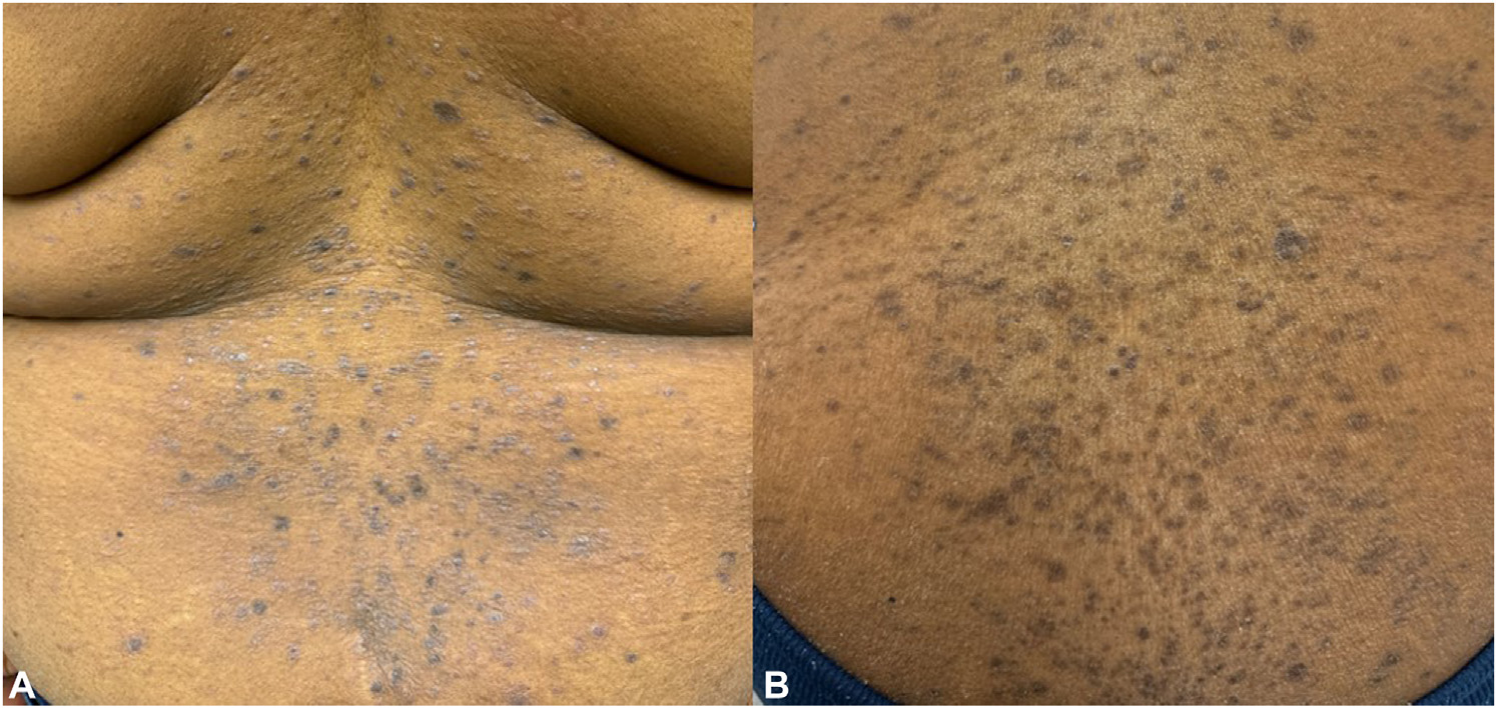
**A** and **B**, Generalize cutaneous lichen planus (LP) presenting as innumerable pruritic, shiny, violaceous, flat-topped papules (Fig 5, *A*) that evolved into post-inflammatory hyperpigmentation (PIH) and improvement of pruritis following 5 m use of upadacitinib (UPA) (Fig 5, *B*).

A female in her 40s presented with PMH significant for obesity presented with pruritic, innumerable, flat-topped, shiny, violaceous papules on her back (Fig 5, *A*) for 5 m duration. PNRS was 10 out of 10. A biopsy was obtained from the back, supportive for generalized cutaneous LP diagnosis. Treatment with clobetasol 0.05% ointment, hcq 200 mg, and MMF 1000 mg, all twice a day for 4 m were attempted and improved the PNRS to 7 out of 10, but the LP still adversely affected her QoL. The hcq and MMF were switched to UPA 30 mg daily for 2 weeks and 15 mg daily for 5 m. PNRS improved to 3 out of 10 and the majority of the lesions have evolved into PIH (Fig 5, *B*) and she is happy with maintenance dosing 15 mg three times a week on 1 year follow-up with continuation of occasional use of the clobetasol ointment during her worst flares.

## Discussion

UPA is currently FDA-approved in dermatology for atopic dermatitis. It is a selective JAK1 inhibitor. LP and LPP are diverse inflammatory conditions that can have significant impact on QoL. They can be recalcitrant to traditional treatments; those treatments often do not provide sustained control. The efficacy of UPA can be attributed to aberrant JAK-STAT pathway signaling as a contributor of cutaneous LP and LPP pathogenesis, including the JAK1 pathway.^2^^,^^3^ Overexpression of IL-4, IL-25, IL-13, and thymic stromal lymphopoietin, along with one of their downstream effectors JAK1, was identified in immunohistochemistry of dermal lymphocytic infiltrates of lesional cutaneous and oral LP^2^^,^^3^^,^^4^, ^5^, ^6^ This suggest both Th1 and Th2 imbalances may play a role in cutaneous LP, which partially explains efficacy of UPA in treating LP. UPA is an effective type 2 inflammation regulator, shown by its great efficacy in treating atopic dermatitis. Dupilumab, which almost exclusively regulates the Th2 pathway, was reported to improve cutaneous LP, further supports dysregulation of Th2 pathway as a possible culprit of cutaneous LP.^7^, ^8^, ^9^, ^10^ As JAK1 is also a broad effector molecule of other cytokines and pathway, UPA may also partially regulate other cytokines and pathway not aforementioned previously that contribute to development of cutaneous LP. Abrocitinib, another selective JAK1 inhibitors, is also effective in treating recalcitrant cutaneous LP.^11^ While there are other cases in literature reporting success of using UPA on cutaneous LP (Table I),^6^^,^^11^, ^12^, ^13^, ^14^, ^15^, ^16^, ^17^, ^18^, ^19^, ^20^, ^21^ this case series uniquely highlights rapid and sustained long term improvement even after the doses were lowered. The unique lower dosing regimens may limit potential for side effects. These cases also showcase UPA efficacy on rare and more-difficult-to treat variants.

A phase II randomized clinical trial for cutaneous LP and LPP with secukinumab did not proceed to phase III, thus leaving LP without any systemic treatment for moderate-to-severe disease to-date.^22^ While tofacitinib has shown positive response, its non-specific targeting increases risk of deleterious adverse events. Ruxolitinib 1.5% cream open label trial showed a promising result, but has a body surface area limitation, further highlighting the treatment gaps and needs for effective systemic therapy.^23^ While promising, additional controlled studies are needed to evaluate UPA as a safe and effective treatment option for cutaneous LP and LPP.

## Conflicts of interest

Dr Tjahjono has served as a consultant and speaker for Arcutis, Bristol Myers Squibb, Eli Lilly, Incyte, and Galderma. Dr McNamara has no conflicts of interest to disclose.
